# Turkish women’s opinions about cesarean delivery

**DOI:** 10.12669/pjms.306.5748

**Published:** 2014

**Authors:** Rukiye Hobek Akarsu, Salime Mucuk

**Affiliations:** 1 Rukiye Hobek Akarsu, RN, Msc, Research Assistant, Nursing Department, University of Bozok, School of Health, Yozgat, Turkey.; 2 Dr. Salime Mucuk, Assistant Professor, Nursing Department, University of Erciyes, Health Sciences Faculty, Kayseri, Turkey.

**Keywords:** Cesarean rate, reasons for cesarean delivery, preference of delivery mode, satisfaction with cesarean delivery

## Abstract

***Objective:*** This study explores the opinions of the Turkish women about cesarean delivery and the reasons and factors affecting their preference for it.

***Methods:*** This is a descriptive study conducted at Maternity Hospitals in Yozgat, Turkey. A total of 423 women who were on the first cesarean day participated in the study. Data were collected through a questionnaire form administered in the form of a face-to-face interview. Pearson Chi-Square test and logistic regression analyses were used for the statistical analyses.

***Results:*** The findings of the study revealed that 13 percent of the participating women reported that the ideal mode of delivery was cesarean delivery while a great majority (87%) opted for vaginal delivery. About 53% of them stated that they would prefer cesarean section for their next delivery while 47% reported that they would choose vaginal delivery . It was observed that 72.1% of participants were satisfied with cesarean delivery; 44.4% of the women were found to prefer cesarean delivery due to labor pain and fear.

***Conclusion:*** It was determined that nearly half of the women preferred cesarean delivery due to labor pain and fear despite the fact that they believe that the ideal mode of delivery is vaginal delivery.

## INTRODUCTION

One of the most significant factors influencing a healthy process and termination of pregnancy is the mode of delivery. Although cesarean delivery, when necessary, is a safe method for both the mother and the baby, it is emphasized that maternal mortality and morbidity rate in cesarean delivery is higher than vaginal delivery.^1^^,^^2^ Recently, there has been a clear increase in the rate of cesarean delivery in the developed and developing countries. According to the 2010 figures of World Health Organization (WHO), the rate of cesarean delivery is 30.2% in the United States, 22% in England, 37.4% in Italy, and 41.3% in Brazil.^3^^,^^4^ The lowest rate of cesarean delivery among the developed countries is in Holland (13.7%), in Sweden (16.5%) and in Czechoslovakia (18.4%).^4^ According to the 2008 report of Turkey Population Health Research (TPHR), 45% of the babies were born through cesarean delivery. This indicates that there has been a 24% increase in cesarean delivery since 2003, with every one of two babies born through cesarean section.^5^^,^^6^ These data demonstrate that the rate of cesarean delivery in Turkey is much higher than the WHO-recommended rate (15%) and has gradually been increasing.^7^

Women who give birth by cesarean delivery are subject to anesthesia-related complications. Cesarean delivery also causes a delay in both mothers’ recovery and mother-baby interaction. Thus, increased medical care needs of mothers and babies following the cesarean delivery result in high economic costs.^1^

The determination of women’s opinions about cesarean delivery and the underlying factors behind their choice in delivery mode can significantly contribute to the specification of the measures to be taken to reduce the rate of cesarean delivery as well as preventing its negative consequences. This study explored women’s opinions about cesarean delivery, reasons for opting this and the factors for their preference for cesarean.

## METHODS

The study was conducted at Maternity Hospitals in the city of Yozgat in Turkey. There are two (a public and a private) maternity hospitals in the city. Women can deliver their babies in whichever one they want. Out of 2351 women who gave birth through cesarean delivery between April and December 2011 in Yozgat maternity hospitals, the sample included 423 women (195 women in state hospital and 228 women in private hospital) chosen for an obvious population with a 71.1% of labor pain and labor fear determined by Gozukara and Eroglu with 5% fallibility and 80% power.^2^ The study involves 423 voluntary women who stayed at the postpartum care clinics of the above mentioned hospitals on their first post-operation day.

The data were collected by means of a questionnaire administered in the form of a face-to-face interview. The interviews took place in the participating patient’s room and lasted 15 to 20 minutes. The questionnaire included 38 items regarding women’s socio-demographic characteristics (age, educational status, and employment), Obstetrics characteristics (gravida, previous mode of delivery, spontaneous or IVF pregnancy, pregnancy follow-up hospital), cesarean delivery indication, reason for preferring cesarean delivery, and their preferences for the next delivery. 

Ethical approval was obtained from the Institutional Review Board of Erciyes University. Written official approvals for the research were obtained from the Administrative Units of the Maternity Hospitals and Health Directorate of Yozgat Province. The participants’ written and oral consents were obtained following their being informed about the purpose of the study. For the statistical analyses, percentages and Chi-square tests were used. For the data which were found to be significant in chi-square test, subsequent logistic regression analyses were performed. Significance was set at 0.05.

## RESULTS

Mean age of the women was 26.6**±**5.2 years. 72.1% of the women were found to be satisfied with the cesarean delivery. While a great majority of the women (87 percent) stated that the ideal mode of delivery was vaginal delivery, the others (13 percent) reported that cesarean delivery was the ideal option. 53% of the participants stated that they would prefer cesarean delivery for their next delivery. In addition, 13.4% them told that the choice of cesarean delivery was their own decision while 68.6% reported that it was their doctors’ preference (Table-I).

Regarding the reasons for preferred cesarean delivery, the findings demonstrated that 44.4% of the women chose cesarean delivery due to the labor pain and labor fear, 14.8% because of repeated cesarean delivery, and 14.8% as a result of their belief that cesarean delivery is healthier for the baby. Women who preferred vaginal delivery stated that vaginal delivery is healthier and more natural (65.5%), and involves shorter postpartum healing process (19.2%) (Table-II).

**Table-I:** Thoughts of women for cesarean delivery (n = 423).

	***n***	***%***
Satisfaction with cesarean delivery		
Satisfied Not satisfied	305 118	72.1 27.9
Ideal delivery mode		
Vaginal delivery Cesarean delivery	368 55	87.0 13.0
Believed delivery mode		
Vaginal delivery Cesarean delivery	261 162	61.7 38.3
Preferred delivery mode for next pregnancy		
Vaginal delivery Cesarean delivery	199 224	47.0 53.0
Who decided to cesarean delivery		
Herself /husband/family Doctor Doctor and herself	57 290 76	13.4 68.6 18.0
Explanation of causes of cesarean delivery		
Explained Not explained	346 77	81.8 18.2
Total	423	100

**Table-II:** Reasons’ for preferred cesarean or vaginal delivery.

***Reasons*** *** for preferred cesarean delivery*** ***(n=162)***	***n***	***%***
Fear/pain of vaginal delivery	72	44.4
Repeat cesarean	24	14.8
More healthy for the baby	24	14.8
Tubal ligation	16	9.9
Cephalopelvic disproportion	15	9.3
Chronic diseases	11	6.8
**Reasons** ** for preferred vaginal delivery** **(n=261)**		
More natural and healthy	171	65.5
Shorter postpartum healing process	50	19.2
Postpartum painless	40	15.3

**Table-III:** Preferred delivery mode according to descriptive characteristics of women (n=423).

***Descriptive Characteristics ***	***Preferred delivery mode***						
	***Vaginal***		***Cesarean***		***Total***		***x*** ^2 ^ ***/p***
	***n***	***%***	***n***	***%***	***n***	***%***	
Age (years)							
18- 22	72	75.0	24	25.0	96	100.0	
23- 27	93	58.1	67	41.9	160	100.0	x²=11.618
28- 32	61	58.1	44	41.9	105	100.0	p=0.020
33- 37	29	1.7	18	38.3	47	100.0	
38- 42	6	40.0	9	60.0	15	100.0	
Education							
Illiterate	88	62.9	52	37.1	140	100.0	
Primary school	96	68.1	45	31.9	141	100.0	x²=5.939
High School	49	55.1	40	44.9	89	100.0	p=0.115
University	28	52.8	25	47.2	53	100.0	
Employment							
Employed	24	49.0	25	51.0	49	100.0	x²=3.212*
Unemployed	237	63.4	137	36.6	374	100.0	p=0.073
Total	261	61.7	162	38.3	423	100.0	

* *Yates correction was made.*

**Table-IV:** Preferred delivery mode according to obstetrics characteristics of women (n = 423).

***Obstetrics characteristics***	***Preferred delivery mode***						
	***Vaginal***		***Cesarean***		***Total***		***x*** ^2 ^ ***/p***
	***n***	***%***	***n***	***%***	***n***	***%***	
Gravida							x²=0.016 p=0.898
Primigravida	74	62.2	45	37.8	119	100.0	
Multigravida	187	61.5	117	38.5	304	100.0	
**Previous mode of delivery(n=277)**							
Vaginal delivery	38	50.0	38	50.0	76	100.0	x²=6.688 p=0.083
Cesarean delivery	113	67.3	55	32.7	168	100.0	
Vaginal and cesarean delivery	21	63.6	12	36.4	33	100.0	
Pregnancy							x²=0.000 * p=1.000
Spontaneous	252	61.6	157	38.4	409	100.0	
IVF	9	64.3	5	35.7	14	100.0	
**Pregnancy follow up** ** (n=422)**							x²=15.255 p=0.000
State hospital	169	69.5	74	30.5	243	100.0	
Private/ University hospital	91	50.8	88	49.2	179	100.0	
Delivered at							x²=8.678 p=0.003
State hospital	135	69.2	60	30.8	195	100.0	
Private/ University hospital	126	55.3	102	44.7	228	100.0	
Total	261	61.7	162	38.3	423	100.0	

* *Yates correction was made*

**Table-V:** Women's age group and the birth hospital effect on the delivery mode decisions: logistic regression analysis results (n =423).

***Variables ***	***B***	***Wald***	***p***	***Exp(B)***	***%95.0 CI for EXP(B)***	
					***Lower***	***Upper ***
The birth hospital (Ref. State hospital)	.564	7.512	.006	1.758	1.174	2.633
Age (year)	.041	4.398	.036	1.042	1.003	1.083
Constant	-1.892	12.006	.001	.151		

Twenty five percent of the women aged between 18 and 22 preferred cesarean delivery, but for the women aged between 38 and 42, this percentage was higher (60%). The difference between age groups in terms of preferred delivery mode was statistically significant (p=0.020) (Table-III). It was observed that women’s preference for cesarean delivery increased with advanced age. However, no statistically significant difference was observed in the preferred delivery mode in terms of educational status (Table-III).

There were no statistically significant differences between the primigravida and multigravida (p=0.898) and between spontaneous and IVF pregnancy (p=1.000) in terms of preferred delivery mode. It was determined that previous delivery mode did not affect the preferred delivery mode (p=0.083). The results also indicated that 49.2% of the pregnant women followed up at private hospital and 30.5% of them who were followed up at public hospitals preferred cesarean delivery. These differences were statistically significant (p=0.000) (Table-IV). It was discovered that the rate of preference for cesarean delivery was 1.75 times higher for the pregnant women who gave birth at private hospital than those who wanted to give birth at public hospitals (Table-V).

## DISCUSSION

The findings indicated that the women’s preference for cesarean delivery was due to: labor pain and fear (44.4%), the fact that cesarean was their previous mode of delivery (14.8%), the opinion that it would be healthier for the newborn (14.8%), the wish to have tubal ligation and cesarean delivery at the same time (9.9%), the pelvic dystocia (9.3%), and having a chronic disease (6.8%) (Table-II). When the rates of the actual reasons for the cesarean section of the women were investigated, it was seen that 16.1% of the women had real indications for cesarean section, with 9.3% with pelvic dystocia and 6.8% with a chronic disease whereas the remaining group (83.9) had the potential to give birth by vaginal delivery. Reported by nearly half of the participants, fear of pain constituted the most common reason for the women’s preference for cesarean delivery. In line with these findings, the study of Torloni et al. reported that the main reason for preferring cesarean delivery was fear of pain.^8^ Such high rates of cesarean delivery in Turkey will be reduced if the appropriate measures are taken to eliminate the negative effects of the fear of labor pain. Health care personnel and pregnant women should be trained about labor pain, labor fear and coping methods, and implementing these methods may help women efficiently manage the labor pain and fear. Another reason stated by the women for their preference for cesarean delivery was the fact that their previous delivery was cesarean (14.8%) and that they thought it would be healthier for the newborn (Table-II).

These findings are in contradiction with the fact that the success of the vaginal delivery after a cesarean section has been increasing across the world. 19.9% of the women in the United States, 5.7% in Norway, 53% of the women in Sweden were recommended to get vaginal delivery after cesarean delivery.^9^ In the previous studies, the most common indication for the cesarean delivery was having a previous cesarean section, reported by between 25% and 38% of the participating women.^10^^-^^14^ Gozukara and Eroglu, reported that 71.1% of the women preferred cesarean delivery due to labor pain and fear, 15.5% because of their belief that cesarean delivery was healthier, and 11.1% on account of having health problems.^2^ Caglayan et al. found that 34.8% of the women received cesarean section due to having a previous cesarean delivery, 27.8% because of dysfunctional labor, 17.6% as a result of fetal distress, and 8.7% presenting transverse position along with 11.1% presenting other indications.^10^ These results are similar to those of the current study.

Another significant finding obtained in the current study was that 87% of the women thought that the ideal delivery mode was vaginal delivery, with only 47% of the women reporting that they wanted vaginal delivery for their next delivery. In light of these findings, it may be argued that although women regard vaginal delivery as the ideal mode of delivery, they prefer cesarean delivery in practice. Besides all these, the rate of satisfaction with cesarean delivery was rather high (72.1%) (Table-I). 

 The results obtained from this study demonstrated that 86.6% of the choice of the delivery mode was made by the doctors and the women themselves whereas only 13.4% of was made by the women themselves with the help of their family (Table-I). In line with these findings, another study revealed that it was the doctors who decided the mode of delivery for more than half of the mothers.^2^^,^^8^ It seems quite normal that women leave the choice of delivery mode to the doctors who are supposed to make the most proper decisions both for their health and their babies. Nevertheless, the rate of the actual reasons for the cesarean section of the women was lower (16.1%, Table-II).

The findings of this study also showed that 89.1% of the cesarean sections were performed during day shift, which indicates that delivery time was not determined according to the normal course of the birth but was planned rather than coincidental. The cognition that following up the woman continuously during the labor process is a time-consuming and hard task can be a reason for doctors’ opting for cesarean section.

The rate of cesarean delivery at private hospitals was found to be 1.7 times higher than state hospitals (p<0.05) (Table-V). These findings are in line with the previous research which emphasized that giving birth at a private hospital increased the rate of cesarean delivery.^15^^,^^16^ We are of the opinion that women in private hospitals have an opportunity to express their wishes more freely and comfortably than in state hospitals, which leads them to prefer the private hospitals where their wishes are more readily welcomed.

The results of this study indicated that there is a positive correlation between the age of the women and the rate of the choice of cesarean delivery (p<0.05) (Table III and V). Similarly, Fuglenes et al. reported that women’s preference for cesarean delivery increased with advanced age.^17^ Advanced age pregnancy has been increasing due to higher rates of women’s labor force participation, marriage at a later age, and advanced health care opportunities. It can be argued that relatively higher risks of pregnancy, labor and postpartum complication in advanced age increase women’s preference for cesarean delivery.

 The findings of the current study also revealed that educational and employment statuses of the women did not affect the choice of delivery mode (p>0.05) (Table-III), which bears similarity to the study of Sayiner et al. in which income and employment status of the women were found to have no effect on their preference regarding the type of delivery.^18^ Likewise, Angeja et al. reported that educational status of the women did not affect preferences for the delivery type.^19^ Unlike the findings of this study, some other studies indicated that women with higher educational status preferred cesarean delivery more than those with lower educational status.^20^^,^^21^ The sample of this study included a small number of women who were employed and who had high educational level, which might have affected the results.

## CONCLUSION

The findings of this study indicated that nearly half of the participating women preferred cesarean delivery due to labor pain and fear although they believe that the ideal mode of delivery is vaginal delivery.
